# An integrative approach to understanding diversity patterns and assemblage rules in Neotropical bats

**DOI:** 10.1038/s41598-023-35100-z

**Published:** 2023-06-01

**Authors:** María A. Hurtado-Materon, Oscar E. Murillo-García

**Affiliations:** 1 Department of Ecology and Conservation Biology, Texas A&M University, College Station, TX 77843 USA; 2 Grupo de Investigación en Ecología Animal, Departamento de Biología, Universidad del Valle, 760001 Cali, Colombia

**Keywords:** Biodiversity, Community ecology, Conservation biology, Evolutionary ecology, Tropical ecology

## Abstract

Understanding the mechanisms shaping species composition of assemblages is critical for incorporating ecological and evolutionary perspectives into biodiversity conservation. Thus, we quantified the relative support of community assembly mechanisms by assessing how species richness relates to the functional and phylogenetic biodiversity of Neotropical bat assemblages. We assessed the association of functional diversity for functional categories and phylogenetic diversity with species richness for 20 assemblages of Neotropical bats. In addition, we contrasted functional and phylogenetic diversity against null models to determine the mechanisms that structure the assemblages. We hypothesize functional/phylogenetic overdispersion for high species sites and a positive relationship between those dimensions of diversity and richness. Functional divergence increased with species richness, indicating that the variability in ecological attributes among abundant bats increases as the assemblages contain more species. Taxa were more distantly related as richness increases, but distances among closely related species remained constant. We found a consistent tendency of clustering of functional traits in site assemblages, particularly in abundant species. We proposed competition between clades as a possible mechanism modulating the community structure in Neotropical bat assemblages. Our results suggest that decreasing overlap in functional traits between abundant species could promote coexistence with rare species that can buffer ecosystem function due to species loss.

## Introduction

Understanding the processes that determine the variety of coexisting species in natural communities is a challenge for ecology, and it is vital in assessing the effects of anthropogenic disturbances and improving conservation strategies^1^. Ecological processes that contribute to community assembly include biotic interactions^2^, environmental conditions^3^, and limited dispersal coupled with demographic stochasticity^4^. Thus, ecological processes shape patterns of functional (distribution of traits) and phylogenetic (evolutionary relatedness) diversity among co-occurring species in assemblages^5^. Reaching an entire understanding of community ecology requires unraveling the relative importance of assembly processes for natural communities by integrating the multiple dimensions of diversity (taxonomic, functional, and phylogenetic)^6^.

Functional traits and phylogeny offer different, and often complementary, information about differences between species^7^. The functional dimension of diversity measures those components of biodiversity that influence ecosystem functioning^8^. The phylogenetic dimension measures evolutionary relationships between taxa^9^ to reflect how much evolutionary history is behind the species constituting the communities^6^. Patterns of functional and phylogenetic diversity are the result of species responses to ecological processes (e.g., environmental conditions, biotic interactions) through the magnitude of trait variation and phylogenetic relatedness among co-occurring species, respectively^10^.

It is possible to assess the relative importance of the mechanisms that structure communities by (1) evaluating the change in species traits (functional diversity) and phylogenetic distances (phylogenetic diversity) with richness across sites, and by (2) comparing local diversity (phylogenetic or functional) with random distributions^11^. If there is phylogenetic conservatism of traits, then the functional and phylogenetic diversity will have the same association with richness and null model comparisons. Competition and niche partitioning mechanisms produce similar patterns between diversity metrics, richness, and null model comparisons. These patterns are an increase in functional/phylogenetic diversity with an increase in species richness and functional/phylogenetic overdispersion. Functional/phylogenetic overdispersion occurs when there is more dispersion than expected under a null model^5^. A decrease in diversity dimensions as species richness increases and as functional/phylogenetic clustering occurs may arise when abiotic filters or inter-clade competition restricts particular traits of species^5,12^. There is empirical evidence supporting these mechanisms for animal and plant communities^5^. However, there is no consensus on which processes generate the current biodiversity patterns. Thus, understanding community assembly mechanisms require conceptual integration of the multidimensional nature of biodiversity through the accumulation of empirical information on functional and phylogenetic diversity patterns of diverse and ecologically important assemblages^13^.

Bats are crucial for Neotropical ecosystems, playing essential roles as insect pest controllers, and contributing to forest maintenance and restoration through the ecological processes of pollination and seed dispersal^14^. Therefore, bats present high local species diversity, broad dietary habits^14^, and great mobility^15^. Given that most species belong to the Neotropical leaf‐nosed bats (Phyllostomidae family), there is a close phylogenetic relationship between bat communities ^14^. They form local guilds with high species richness and a high potential for interspecific competition^16^. In phyllostomids bats, dietary specializations during their radiation imposed functional demands that have influenced cranial evolution, which is associated with skull morphology, bite force, and diet^17,18^. This relationship suggests strong clade-based links between ecological opportunity and diversification^19^. Thus, studying the diversity patterns of Neotropical bats can help understand the mechanisms driving community assembly in highly diverse assemblages.

The lack of conceptual integration of the multifaceted nature of biodiversity and of the possibility to evaluate it in a natural gradient has restricted the understanding of the spatiotemporal dynamics that regulates the patterns of diversity^13^. To increase the understanding of the processes that drive Neotropical community diversity, we assessed the relative contribution of community assembly processes on Neotropical bat assemblages. Specifically, we assessed the relationship between species richness and diversity (functional and phylogenetic) and compared the values of local functional and phylogenetic diversity with the expected value from null models. We aim to describe the patterns of functional and phylogenetic diversity with the species richness variation and to compare observed diversity values with random distributions to determine the possible mechanisms that modulate community structure. We made our predictions based on phylogenetic conservatism of traits. We predicted that (1) functional and phylogenetic diversity would increase with species richness and (2) functional/phylogenetic overdispersion for high species sites indicating that either competition or niche partitioning is the primary community assembly mechanism.

## Results

### Taxonomic coverage

We measured functional traits for 1871 individuals of 97 species (32 genera and six families) in the 20 study sites, with most of the species (80%) belonging to the Phyllostomidae family (Vespertilionidae 10%, Emballonuridae 4%, Mollosidae 4%, and Noctilionidae and Thyropteridae with 1%). Local species richness varied widely (mean 20.15 ± 9.33 species) from five species in Finca Bengala (Salento, Quindio, 2295 m above sea level) to 43 species in Bajo Calima (Valle del Cauca, 220 masl). The species with most of the records were *Dermanura rosenbergi* (208 individuals), *Carollia perspicillata* (152 individuals), *Artibeus lituratus* (147 individuals), *D. rava* (115 individuals), *C. brevicauda* (94), *Sturnira erythromos* (81 individuals), and *Platyrrhinus dorsalis* (79 individuals). On the other hand, *A. amplus, Eptesicus brasiliensis, Eumops glaucinus, Histiotus humboldti, Lonchophylla thomasi, Myotis albescens, Phyllostomus latifolius, P. branchycephalus, Rhogeessa io*, and *Trachops cirrhosis* were rare, represented by one capture per species.

### Relationships between species richness and functional and phylogenetic diversity

**Figure 1:**
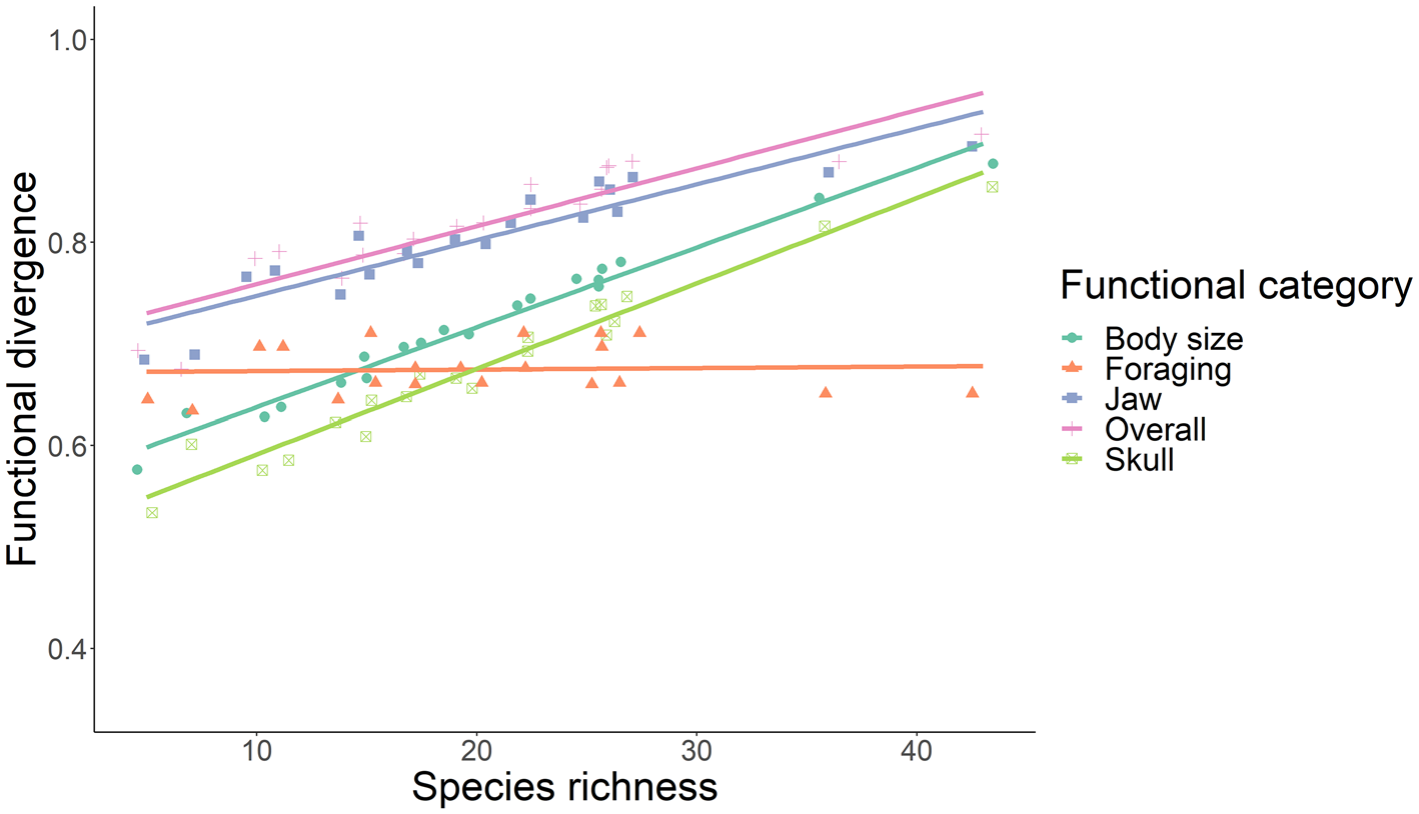
Effects of species richness on functional divergence in Neotropical bat assemblages across Western and Central Mountain ranges of Colombian Andes based on a Generalized Linear Mixed Model.

**Figure 2:**
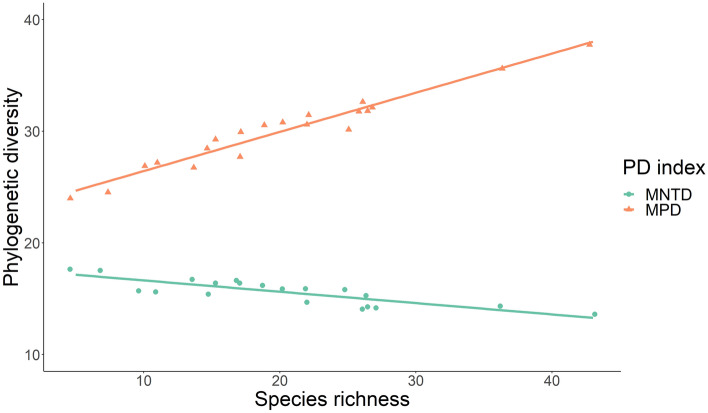
Effects of species richness on phylogenetic diversity in Neotropical bat assemblages across Western and Central Mountain ranges of Colombian Andes based on a Generalized Linear Mixed Model. MPD: mean phylogenetic distance, MNTD: mean nearest taxon phylogenetic distance.

We found that the most abundant species diverge in their ecological function as species richness increases, indicated in the positive relationship between species richness and the functional divergence for body size, jaw, skull, and overall (Fig. 1). However, we did not find any significant association between species richness and functional dispersion, evenness, and uniqueness for any functional category (Supplementary Figs. S1, S2, S3). We found that greater number of species are associated with greater phylogenetic distances, but that the distance between sister taxa did not change as species richness does. This was reflected in a positive association between species richness and Mean Phylogenetic Distance (MPD), but a lack of association between species richness and Mean Nearest Taxon Phylogenetic Distance (MNTD) (Fig. 2). All Generalized Linear Mixed Models converge based on R-hat and visual inspection of the chains of MCMC method, whereas Bayesian p-values (0.025 ≤ *p* ≥ 0.975) and X^2^ discrepancy measure (lack of fit ≈ 1.0) indicated a good fit of models to the observed data (Supplementary Table S1).

### Null model comparations

**Figure 3:**
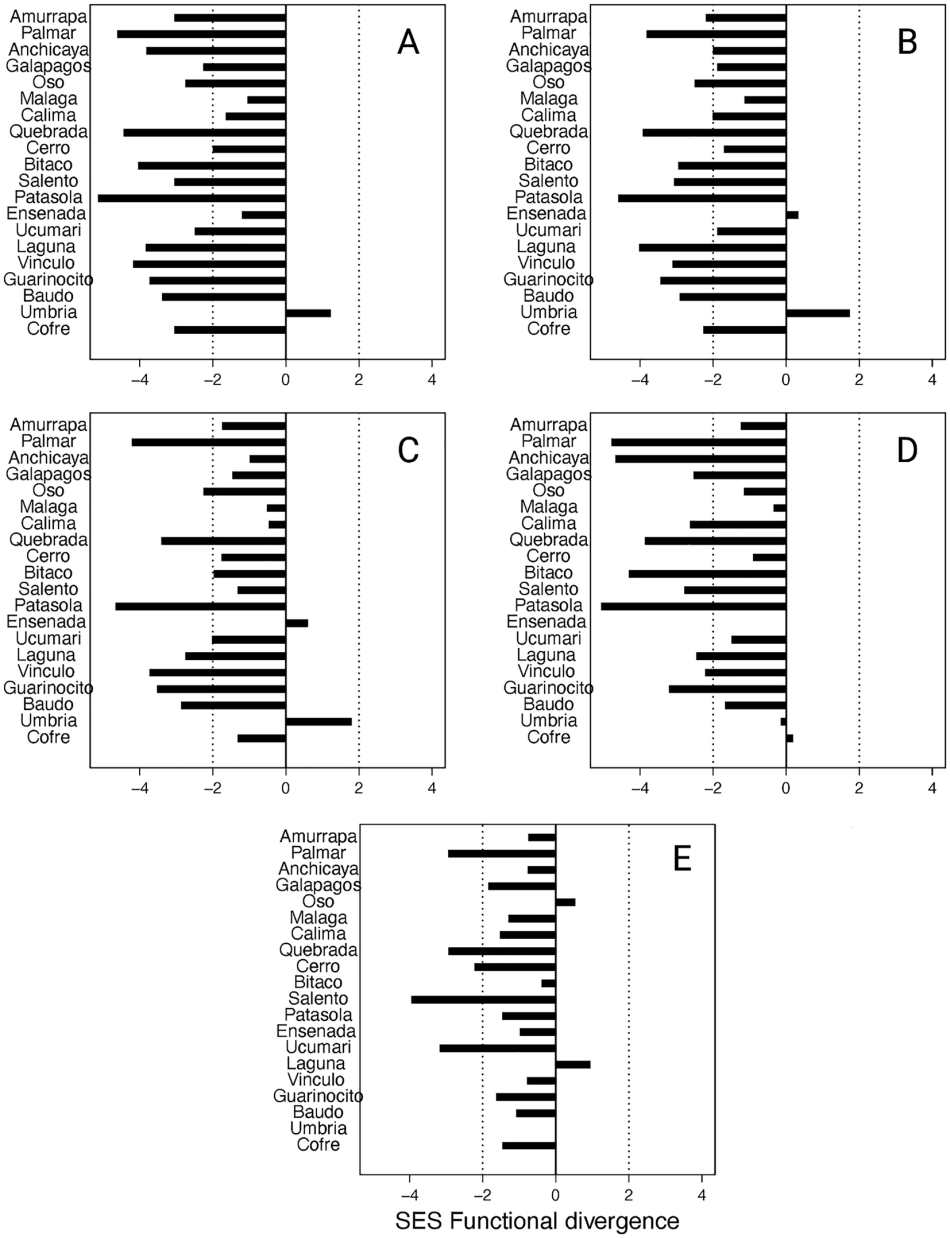
Standardized effect sizes (SSE) of functional divergence of different functional categories for assemblages of Neotropical bats across Western and Central Mountain ranges of Colombian Andes. (**A**) Overall, (**B**) Skull, (**C**) Jaw, (**D**) Body size, and (**E**) Foraging. SES values > 2.0 indicate that the community has more divergence than expected by chance, which suggested trait segregation. SES values < − 2.0 suggests trait clustering.

**Figure 4:**
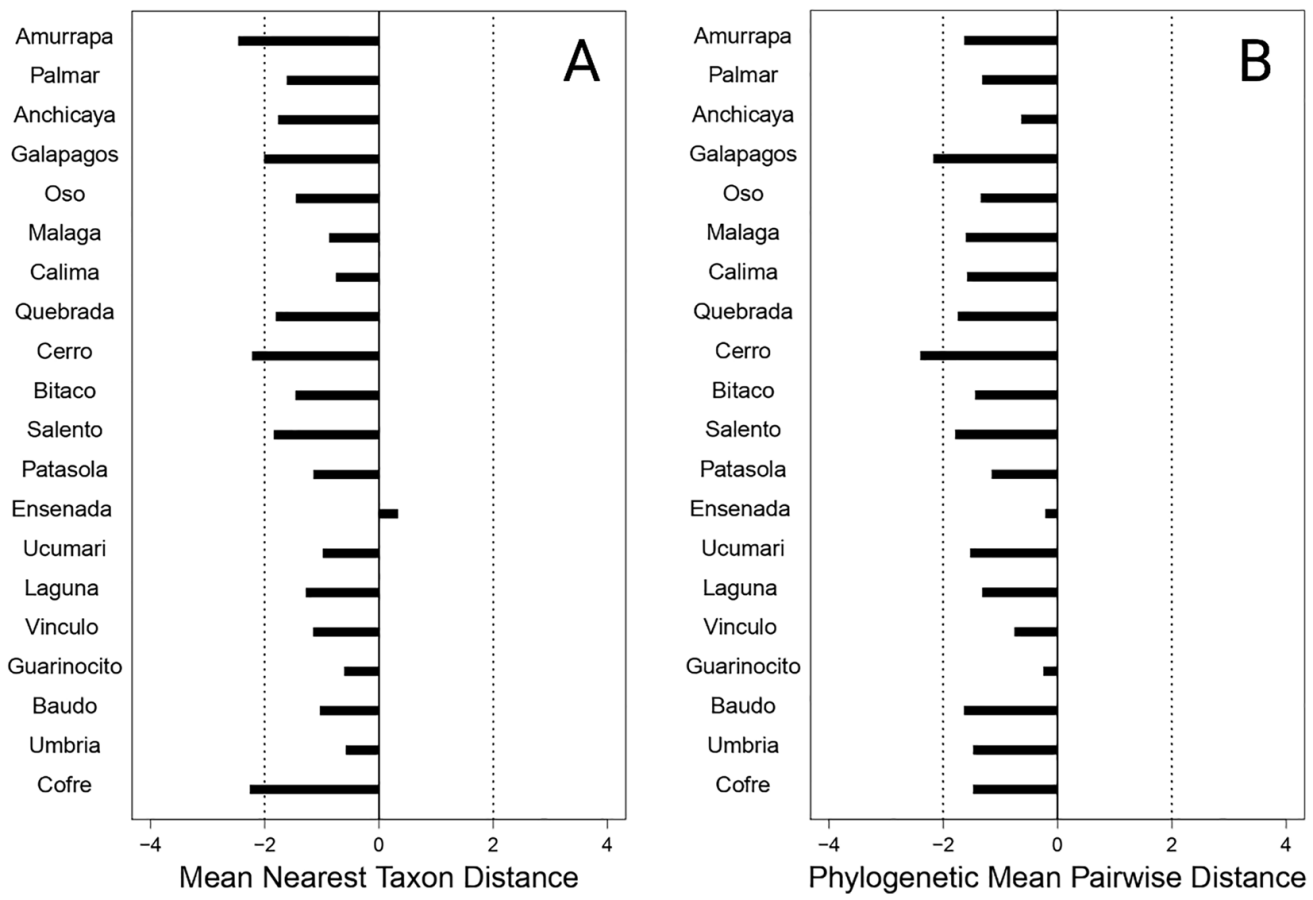
Standardized effect sizes of phylogenetic diversity indexes for assemblages of Neotropical bats across Western and Central Mountain ranges of Colombian Andes. (A) Mean nearest taxon distance (MNTD), and (B) Mean pairwise distance (MPD). SES values > 2.0 indicate that the community has more divergence than expected by chance, which suggested segregation. SES values < − 2.0 suggest clustering.

Our results indicate that the distribution of functional traits tended to be more clustered than expected at random among the most abundant species of bat assemblages. The values of local functional divergence and uniqueness for all functional categories (i.e., body size, skull, jaw, foraging, and overall) were lower than the expected under null distributions (SES < − 2.0) for at least 50% of study sites, except for foraging (< 25%) (Fig. 3). This pattern was stronger for overall (80%) and skull (70%) functional categories. For functional dispersion, less than 50% of study sites showed lower values of SES than expected under the null distributions for all functional categories, except for the foraging category (80%). Very few study sites showed either higher or lower observed values than expected under the null distribution (< 23%) for functional evenness. The distribution of phylogenetic diversity was not different than what is expected at random since most study sites showed SESs between − 2.0 and 2.0 (Fig. 4).

All traits had a significant phylogenetic signal with a mean Pagel’s lambda of 0.96, ranging from 0.70 (Middle skull width) to 1.02 (M_2_ area) (Supplementary Table S2).

## Discussion

Functional divergence for most trait categories (skull, jaw, body size, and overall) and the mean phylogenetic distance increased with bat species richness; however, the other functional and phylogenetic diversity indices did not show any relationship. Local functional divergence indicated trait clustering for most study sites and trait categories. Nevertheless, the other functional and phylogenetic diversity components did not differ from what was expected at random. Our results suggest an increasing differentiation in ecological function between the most abundant species as the local richness of bat assemblages increases, and that competition between clades may be responsible for bat composition at the site scale.

**Figure 5:**
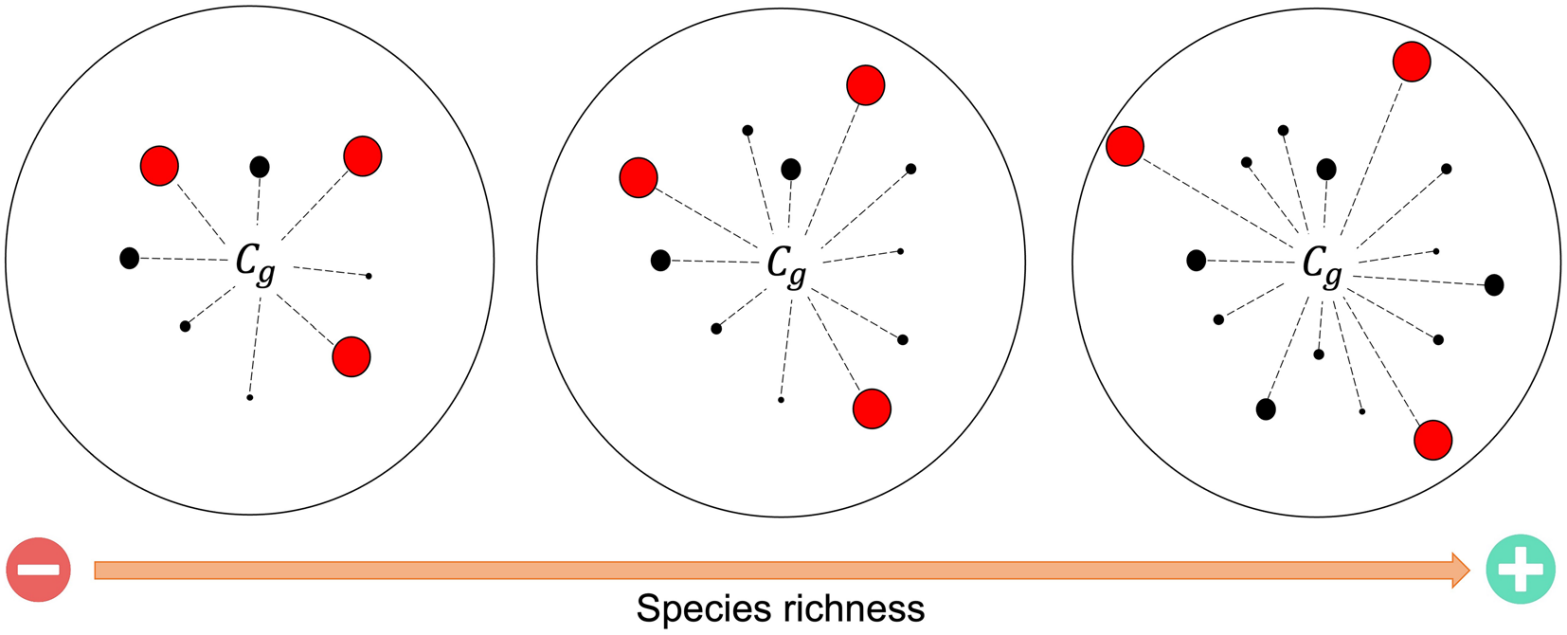
Graphic representation of multidimensional functional space as species richness increases. The distances of the abundant species from the mean of the multidimensional space (centroid) increase with species richness, indicating an expansion in ecological differentiation. Cg is the centroid of the multidimensional space, the circles are the species, and circle size indicates abundance, with abundant species being represented in red.

As expected, we found that the most abundant species are more different in their ecological function as the richness of bat assemblages increases, as indicated by the positive relationship between functional divergence (body size, skull, jaw, and overall) and species richness. Functional divergence represents the spread of trait abundances within the functional space occupied by species of a community^20^. Thus, functional divergence is associated with the degree of niche differentiation among abundant species within communities, with high values indicating that abundant species within communities are very dissimilar, which suggests that those species may compete weakly^21^. On the other hand, and contrary to our expectations, we did not find a relationship between functional dispersion, evenness, and uniqueness with species richness. Consequently, dispersion in trait abundances, the spread of species across trait space, and the functional contribution of single species to overall community redundancy did not increase with richness. Functional overlap does not increase with species richness, even when the most abundant species become more functionally different among themselves. Thus, significant niche differentiation between the most abundant bats may facilitate species’ coexistence as species richness increases, generating functional redundancy among the less abundant species (Fig. 5). This functional redundancy between low abundant species can buffer ecosystem function due to species loss by anthropogenic or environmental disturbances, maintaining the stability of highly diverse ecosystems^22,23^.

Interspecific competition for resources is strong when species have similar traits^24^ causing competitive exclusion or character displacement, which results in trait overdispersion at the community level^25^. However, the probability of interaction is not equal for all species pairs in a community due to different factors^26^. For example, species segregation was particularly evident only for territorial species of understory birds, which face a high interaction potential due to a low dispersal capability among fragments^24^. On the other hand, in natural communities and especially in the tropics, few species are abundant and most are rare^27^. An important consequence of this variation in abundance is that the likelihood of interaction for a given species pair is positively correlated with their abundance at a given site^28^. Hence, while the fraction of abundant species may interact markedly among themselves; the remaining rare species may have fluctuating and erratic interactions with one another. Our results suggest that inside Neotropical assemblages of bats, the species that interact the most are those that are abundant, so they show high functional differentiation as richness increases in these assemblages, which allows the coexistence of ecologically similar and less abundant species. Thus, since competition between rare species may not be strong enough to drive species to local extinction or result in character displacement, it may instead result in coexistence and more similarity in traits than expected under the competition-relatedness hypothesis^29^.

Contrary to our initial predictions, we found functional divergence clustering, which is usually attributed to environmental filters and is expected to be observed in communities inhabiting sites with a stressful climate that restricts the particular trait range in each site^3^. However, we found this clustering of traits for bat assemblages across an altitudinal range from 0 to 3500 m that includes a high variation in environmental conditions. Alternatively, clustering patterns can originate from other processes than environmental filtering^12^. Clade competition can generate these patterns as well if the members of an entire clade that share particular phenotypic traits due to phylogenetic closeness have an advantage, and consequently exclude species from other groups^3,12^. Based on our results, species are equally spaced across the phylogeny in the community regardless of species number (positive relationship between MPD with species richness, but no MNTD); suggesting that the increase in species is through the addition of closely related species. Consequently, our results agree with the hypothesis that clade competition can be a process that produces clustering of traits^3^. Although, we could not distinguish the described pattern from that which is produced if the species are lost randomly^30^.

In summary, we found less functional divergence between the most abundant species of local bat assemblages, but functional divergence increased as assemblages contained more species. This pattern suggests that an increasing differentiation in ecological function between the most abundant species is required as more species with similar morphologies or low abundance are added to assemblages. This results in a high local species richness of ecologically similar bats in tropical communities. The possible mechanism for local bat assemblage is competition between clades^12^, which can generate a lower functional and phylogenetic diversity than expected based on species richness. Our results suggest that the most abundant species of assemblages have negative feedback interactions and neutral interactions with the rare species, which allow the coexistence of several rare species regardless of their functional traits. The increased functional divergence with species richness results from a progressive ecological differentiation between the most abundant species with the accumulation of species; it could reduce competition due to the high probability of interaction and overlapping feeding preferences between abundant species.

## Methods

We selected our study sites from localities represented at the mammal collection of Universidad del Valle (Cali, Colombia) based on the completeness of sampling, which we assessed using the number of visits and the number of specimens collected at each potential site. We selected 20 study sites across the Western and Central ranges of the Colombian Andes, distributed across six of the World Wildlife Fund's Global Ecoregions^31^ and an elevation ranging from 0 to 3670 m above sea level (Table S3). All specimens used in this study were collected by previous researchers and are housed at the mammal collection of Universidad del Valle. Relevant guidelines and regulations for experiments on live vertebrates do not apply.

### Taxonomic diversity

We compiled species richness for each location as a measurement of taxonomic diversity. Hence, we considered only adults and confirmed the identification of all individuals in our study sites by using taxonomic keys for Neotropical bats^32,33^.

### Functional diversity

We compiled categorical and quantitative functional traits that influence the fitness of individuals. We compiled life-history traits from published literature and we measured morphometric traits using a digital caliper with 0.01-mm accuracy. We grouped the traits into functional categories of body size, skull, jaw, foraging, and overall (Table 1). We assumed that the number of individuals collected at each study site is a valid surrogate for species abundance and we evaluated that assumption by comparing our proxy of abundance with captures from other studies performed in some of our study sites or localities close to them^34–38^. We found that the most abundant species reported in those studies were the same as in our study sites, which suggests that the number of individuals deposited in the mammals’ collection is a reliable surrogate for the ranking of bat abundance in our study sites.

**Table 1:** Bat traits and trait categories used to estimate the functional diversity of bat assemblages from the Western and Central mountain ranges of Colombian Andes.

Functional category	Trait	Attribute	Influence about the fitness
Foraging	Feeding strategy^39–41^	Foliage gleaner	Variation of foraging
		Aerial insectivore	
		Nomadic frugivore	
		Sedentary frugivore	
		Sanguivore	
		Piscivore	
		Nectarivore	
		Carnivore o animalivore	
	Wing shape (the ratio of the length of the third digit metacarpal to the length of the fifth digit metacarpal)^42^		Affects flight patterns and thus prey selection
	Relative length of the pinna (ratio of the ear length to the length of de forearm)^42^		Affects echolocation characteristics and thus the detection and capture of prey
Skull	Total skull length^43–45^		Associated dietary differences and protection of the brain and sensory organs
	Length of maxillary toothrow^43–45^		
	Middle skull width^43–45^		
	M2 area^43–45^		
	Breadth across upper molar^43–45^		
	Breadth of braincase^43–45^		
Jaw	Condylo-canine length^43–45^		Associated dietary and feeding strategy differences
	Total length^43–45^		
	Coronoid process height^43–45^		
Body size	Length of forearm^43,46^		Significance in the ecological differentiation in the community and reflect physiological constraints
	Total length^43,46^		
	Mass^43,46^		

We quantified functional diversity by calculating four indexes: functional dispersion^47^, functional divergence, functional evenness^20^, and functional uniqueness^48^. Each functional diversity index was calculated for all study sites and functional categories. We calculated Pagel's Lambda to test whether functional traits exhibited phylogenetic signal^49^.

### Phylogenetic diversity

To calculate phylogenetic diversity, we used the phylogeny of the superfamily Noctilionoidea^50^. For analyses, we substituted the species that were not included in the tree with the closest phylogenetic species of the same genus. We assessed phylogenetic diversity by using the Mean Pairwise Phylogenetic Distance (MPD) and the Mean Nearest Taxon Distance (MNTD) weighted by species abundance^10^.

### Data analysis

We tested the association of species richness with functional and phylogenetic diversity using Generalized Linear Mixed Models. For functional divergence, evenness and uniqueness, we used beta mixed models since these indexes have continuous and bounded values between 0 and 1. On the other hand, for functional dispersion and phylogenetic indexes, we used normal mixed models since index values are continuous and unbounded. To evaluate the potential biases due to differences in sampling effort between site locations, we assessed whether the number of capture days at study sites was correlated with species richness or the number of collected individuals across sites. We found marginally significant associations (species richness/sampling effort Rho = 0.391, *p* value = 0.089; captured individuals/sampling effort Rho = 0.422, *p* value = 0.063), indicating that our measurements of taxonomic diversity are marginally affected by differences in sampling effort among sites, so we included sampling effort (log number sampling days) as a random effect of the model. We added the effect of altitude (m) as a random effect due to its potential influence on species richness. We ran three chains (6,100,000 samples per chain) for an MCMC method to approximate the posterior distribution of model parameters, with the first 100,000 samples used as the burn-in period and a thinning interval of 5000 samples to minimize autocorrelation in the chains. We used a Bayesian *p* and a discrepancy measure based on chi-squared to test for the goodness of fit^51^.

To evaluate whether the dispersion of traits and phylogenetic distances observed at each study site differed from what is expected at random, we compared empirical values of diversity (functional and phylogenetic) with those obtained from a null model. First, we ran 1000 iterations by randomizing species identity and abundance among sites but maintaining species richness for each site and abundance for each species^52^. Then, we calculated the standardized effect size (SES) for each index and each study site to measure the statistical amount of deviation of the observed value from the mean of the null distribution^53^. Finally, we considered that an observed value was not different from a null distribution when its SESs fell between − 2.0 and 2.0^53^. Furthermore, positive SES values > 2 indicate overdispersion (more dispersion than expected), and negative SES < − 2 values indicate segregation (less dispersion than expected) of traits and phylogenetic distances^53^.

We used the statistical language R 4.0.2.^54^ for analyses. We used the dbFD function of the FD package^55^ to calculate functional divergence, dispersion, and evenness; the function uniqueness of the adiv package^56^ to calculated functional uniqueness, and phylosig of the phylotools package to test phylogenetic signal^57^. We used the mpd and mntd functions of the package picante^52^ to calculate MPD and MNTD indices. We randomized community matrices with the randominzeMatrix function of the picante package^52^ and ran generalized linear mixed models in JAGS^58^ with the rjags package^59^. Finally, we graphed our results using the ggplot package^60^.

## Supplementary Information

Supplementary Information 1.

Supplementary Information 2.

Supplementary Information 3.

## Data Availability

All data analyzed during this study are included in this published article (and its appendix file).
