# Supplementary material for: An integrative approach to understanding diversity patterns and assemblage rules in Neotropical bats
Source: Sci Rep. 2023 Jun 1;13:8891. doi: 10.1038/s41598-023-35100-z (PMC10235113; doi:10.1038/s41598-023-35100-z)
Supplement: Supplementary file 3 — Supplementary Information 3. [file 41598_2023_35100_MOESM3_ESM.pdf]

An integrative approach to understanding diversity patterns and assemblage rules in Neotropical bats

Maria A. Hurtado-Materon and Oscar E. Murillo-García

FIGURES

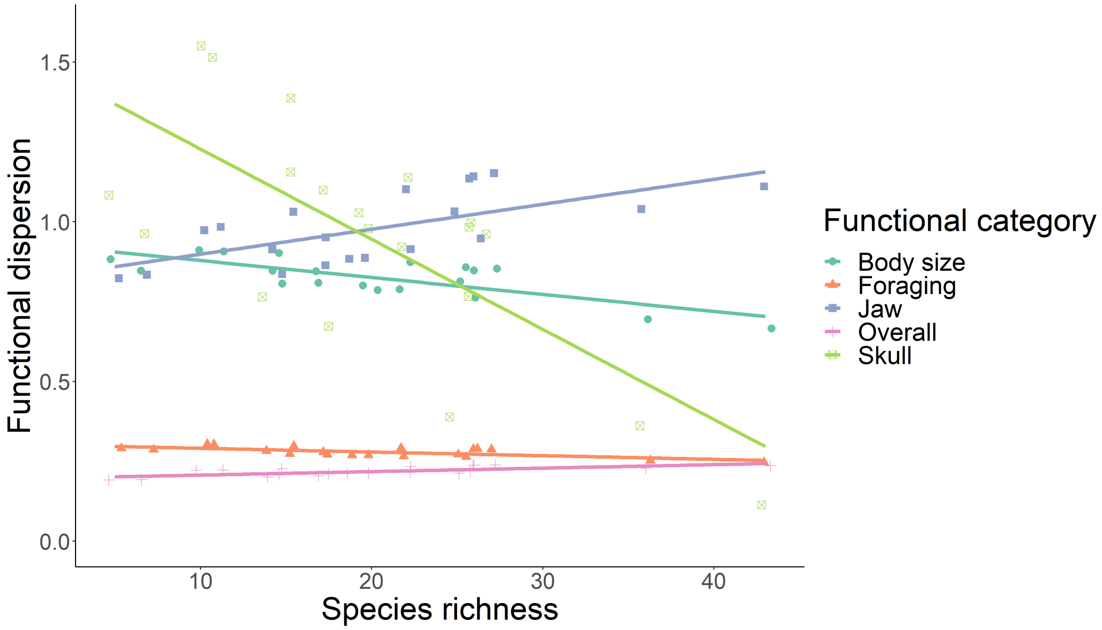

Figure S1. Variation in functional dispersion with species richness for Neotropical bat assemblages across Western and Central Mountain ranges of Colombian Andes.

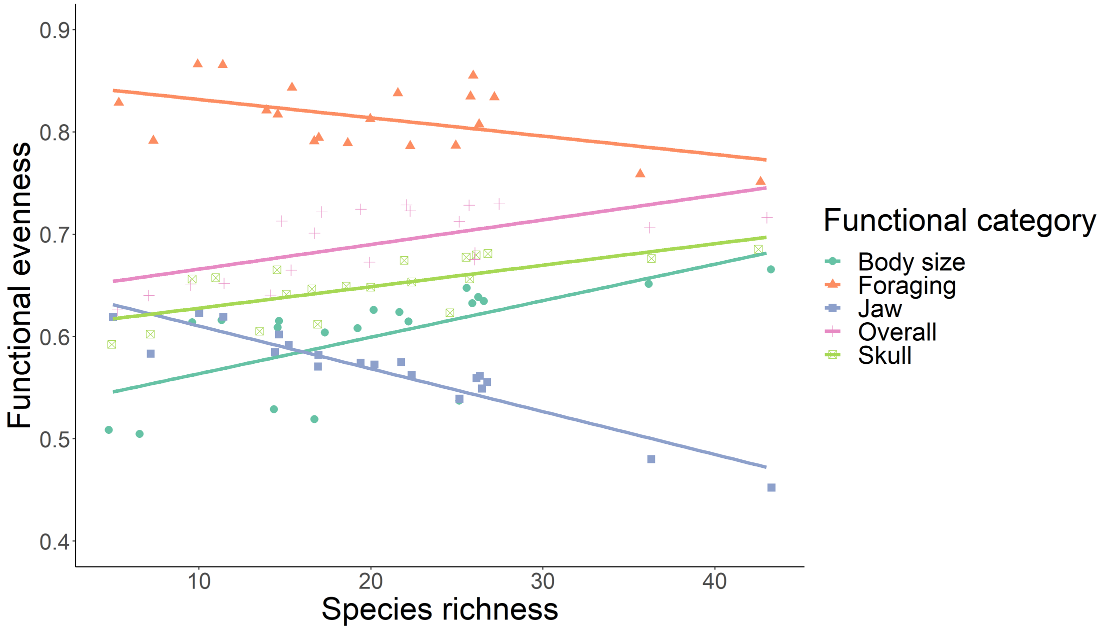

Figure S2. Variation in functional evenness with species richness for Neotropical bat assemblages across Western and Central Mountain ranges of Colombian Andes.

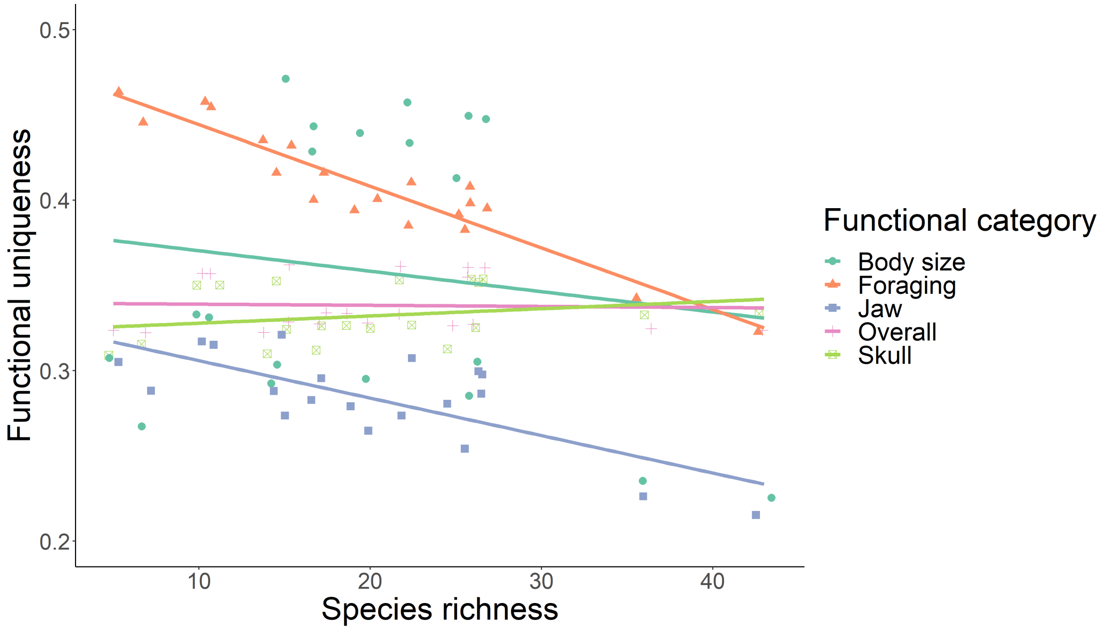

Figure S3. Variation in functional uniqueness with species richness for Neotropical bat assemblages across Western and Central Mountain ranges of Colombian Andes.

TABLES

Table S1. Result Generalized Linear Mixed Models for the relationship between species richness and both functional and phylogenetic diversity in Neotropical bat assemblages.

	**b**	**IC**	**Bayesian p-value**	**Lack of fit**	**b**	**IC**	**Bayesian p-value**	**Lack of fit**
	Functional divergence				Functional dispersion			
Skull	0.04	[0.019, 0.063]	0.561	1.167	-0.035	[-0.088, 0.018]	0.455	1.054
Jaw	0.035	[0.013, 0.057]	0.552	1.175	0.01	[-0.004, 0.025]	0.439	1.042
Size	0.018	[-0.007, 0.043]	0.559	1.177	-0.004	[-0.038, 0.032]	0.454	1.058
Foraging	1.08E-05	[-0.025, 0.026]	0.586	1.236	-0.001	[-0.006, 0.004]	0.448	1.053
Total	0.035	[0.015, 0.056]	0.542	1.168	0.001	[-0.001, 0.003]	0.455	1.054
	Functional evenness				Functional uniqueness			
Skull	0.006	[-0.015, 0.026]	0.513	1.124	0.001	[-0.021, 0.021]	0.504	1.107
Jaw	-0.016	[-0.036, 0.003]	0.51	1.124	-0.009	[-0.032, 0.012]	0.573	1.19
Size	0.009	[-0.017, 0.035]	0.544	1.156	-0.008	[-0.062, 0.044]	0.552	1.18
Foraging	-0.005	[-0.030, 0.023]	0.523	1.13	-0.013	[-0.043, 0.019]	0.564	1.191
Total	0.007	[-0.014, 0.028]	0.504	1.115	-0.001	[-0.018, 0.016]	0.456	1.065
	Phylogenetic diversity							
MPD	0.306	[0.053, 0.575]	0.458	1.053				
MNTD	-0.102	[-0.355, 0.141]	0.454	1.064				

Table S2. Phylogenetic signal analysis results, using Pagel's lambda [54] for functional traits evaluated in Neotropical bat assemblages.

Variable	Lambda	logL	logL0	p-value
Length of forearm	1.007283326	-233.4328652	-252.1063089	9.89E-10
Mass	1.012526458	-267.5122578	-274.2179726	0.000250096
Total length	1.004426824	-263.4624565	-275.2757988	1.17E-06
Wing shape	0.918217529	67.51144412	51.8516141	2.19E-08
Relative length of the pinna	0.84567742	85.64376594	76.05942661	1.20E-05
Total skull length	1.009112078	-196.5644028	-204.8975434	4.46E-05
Breadth of braincase	1.00196301	-112.1240716	-125.4480205	2.44E-07
Length of maxillary toothrow	1.015351782	-124.1632925	-140.6234117	9.60E-09
Breadth across upper molar	1.014563149	-124.667545	-156.7473433	1.15E-15
M2 area	1.020310458	-136.9786198	-162.0337683	1.45E-12
Meddle skull width	0.698406934	-128.2739207	-133.0679473	0.001958475
Total dentary length	0.990595895	-162.4050156	-169.5402938	0.000158322
Condyle-canine length	0.985938147	-160.9227831	-167.8735296	0.000192645
Coronoid process height	1.002440495	-111.750743	-127.389719	2.24E-08

Table S3. Localities across the Western and Central ranges of the Colombian Andes used for this study.

State	Municipality	Locality	Mountain range	Altitude, MASL		Ecoregion
				Mín	Máx	
Valle del Cauca	Buenaventura	Bahía Malaga	Western	0	0	Pacific-South American Mangroves
Chocó	Bahía Solano	Ensenada de Utría	Western	5	10	Chocó-Darien wet forests
Chocó	Bajo Baudó	Piliza	Western	0	100	Chocó-Darien wet forests
Valle del Cauca	Buenaventura	Bajo Calima	Western	40	400	Chocó-Darien wet forests
Caldas	La Dorada	Guarinocito, Hacienda La Española	Central	235	313	Valle del Magdalena montane forest
Risaralda	Pueblo rico	Santa Cecilia, Volga, Alto Amurrapá	Western	413	470	Northeast of the Andes montane forest
Valle del Cauca	Buenaventura	Alto Anchicaya	Western	230	660	Chocó-Darien wet forests
Chocó	San José del Palmar		Western	350	900	Chocó-Darien wet forests
Chocó	San José del Palmar	Alto del oso	Western	1000	1000	Northeast of the Andes montane forest
Valle del Cauca	Buga	El Vínculo	Central	975	1100	Valle del Cauca dry forests
Risaralda	Belen de Umbría		Western	1525	1747	Valle del Cauca montane forest

Cauca	Cajibio	El Cofre, Finca La Herencia	Macizo	1700	1700	Valle del Cauca montane forest
Valle del Cauca	El Cairo	Quebrada Charco azul	Western	1600	1800	Northeast of the Andes montane forest
Valle-Chocó		Alto de Galápagos	Western	1800	2000	Northeast of the Andes montane forest
Valle del Cauca	La Cumbre	Bitaco, Chicoral	Western	1650	2176	Valle del Cauca montane forest
Risaralda	Dosquebradas	Parque Regional Natural Ucumarí, estación Piscicola "El cedral"	Central	2130	2196	Valle del Cauca montane forest
Valle del Cauca	El Cairo	Estación biológica cerro El Ingles	Western	2000	2400	Northeast of the Andes montane forest
Quindío	Salento	Boquía, Reserva Natural La Patasola, finca La Betulia	Central	2200	2600	Valle del Cauca montane forest
Quindío	Salento	Finca Bengala, otros	Central	2740	3250	Valle del Cauca montane forest
Risaralda	Pereira	Laguna de Otún, 6 km N 9 km O (El porvernir)	Central	3150	3560	Valle del Cauca montane forest
